# Investigating the Influence of Infrared Drying Method on Linden (*Tilia platyphyllos Scop.*) Leaves: Kinetics, Color, Projected Area, Modeling, Total Phenolic, and Flavonoid Content

**DOI:** 10.3390/plants9070916

**Published:** 2020-07-20

**Authors:** Kemal Çağatay Selvi

**Affiliations:** Department of Agricultural Machinery and Technologies Engineering, Faculty of Agriculture, University of Ondokuz Mayis, 55139 Samsun, Turkey; kcselvi@omu.edu.tr; Tel.: +90-362-312-1919

**Keywords:** linden leaves, infrared drying, color parameters, projected area, modeling, effective moisture diffusivity, activation energy, total phenolic content, total flavonoid content

## Abstract

The Linden (*Tilia platyphyllos Scop.*) is a highly popular herbal plant due to its central nervous system properties. In this study, thin layer drying kinetics of linden leave samples were experimentally investigated in an infrared (IR) dryer. In order to select the appropriate model for predicting the drying kinetics of linden leaves, eleven thin layer semi theoretical, theoretical, and empirical models, widely used in describing the drying behavior of agricultural products, were fitted to the experimental data. Moreover, the color, projected area (PA), total phenolic content (TPC), and total flavonoid content (TFC) were investigated. The results showed that the drying time decreased from 50 min to 20 min. with increased IR temperature from 50–70 °C. Therewithal, the Midilli model gave the most suitable data for 50 °C, 60 °C. Moreover, Verma et al. and Diffusion approximation models showed good results for 70 °C. The lightness and greenness of the dried linden leaves were significantly changed compared with fresh samples. The PA of dried sample decreased similar to the drying time. In addition, the drying temperature effect on the effective diffusion diffusivity (*D_eff_*) and activation energy (*E_a_*) were also computed. The *D_eff_* ranges from 4.13 × 10^−12^ to 5.89 × 10^−12^ and *E_a_* coefficient was 16.339 kJ/mol. Considering these results, the Midilli et al. model is above the 50 °C, 60 °C, and the Verma et al. and Diffusion to 70 °C, for explaining the drying behavior of linden leaves under IR drying. Moreover, it can be said that the Page model can be used, if it is desired, to express the drying behaviors, partially with the help of a simple equation material by drying. TPC and TFC values were statistically < 0.001 higher in dried samples compared to fresh samples; however, no change has been recorded of TPC and TFC values at different temperatures (50 °C, 60 °C, 70 °C).

## 1. Introduction

The usage of herbal medicinal products and supplements has increased tremendously over the past three decades. This is especially due to their health benefits (as a result of their peculiar chemical composition) [1,2]. Therefore, due to the increasing commercial importance of these products, determination of their drying properties is necessary for preservation and storage in longer periods [3]. From this point of view, Linden (*Tilia platyphyllos Scop.*) is highly popular as a herbal plant because of its central nervous system properties; Linden (Tiliaceae) consists of 44 species [4]. It is also consumed as tea, and known for its tranquilizing and analgesic effects [5]. In addition, fresh herbs (such as linden, thyme, mint etc.) are a splendid source of phenolic components (as flavonoids, phenolic, and others) [6]. Moreover, linden has been considered as diaphoretic although it has not been supported by scientific evidence yet [7]. Moreover, the linden belongs to woody plants that shed their leaves in winter, and their leaves, are rich in a glycoside called Tiliacin, and manganese in ash. Primarily in Turkey, there are only three species in the natural environment: *Tilia tomentosa Moench, Tilia platyphyllos Scop., and Tilia rubra DC* [8].

The most common way used technology can guarantee food safety and develop its quality is through thermal processing. It has not only been successfully applied to food products due to reduction of moisture content to desirable levels, but it also leads to safe storage over a long period [9,10]. In general, conventional drying methods require much time and energy during drying of the products [11,12,13]. It is also reported that some structural properties of the products may be damaged with traditional drying methods, such excessive shrinkage, discoloration, loss of nutrients [14], and severe deterioration of nutritional and sensorial belongings [15].

For this reason, there is a reasonable need to introduce new drying methods to accomplish these problems. Infrared (IR) processing is one of them. To reduce the water content [16], the IR radiation had been implemented in food processing, reducing energy consumption and time spent in the process, securing and ensuring the quality of food stuffs processed [17,18]. That IR is predominantly responsible for the heating effect of the sun is a part of the electromagnetic spectrum [19]. Only if IR is used to heat—or dry—wet products (the radiation penetrates inside the material) does it become heat. The penetration deepness of radiation relies on the properties of the material and wavelength of radiation 3]. In addition, the surface of dried material emits IR radiation without heating the surrounding air. No medium heating is needed between the IR energy source and the material being dried [20]. IR is often more convenient for thin material layers having a large surface subject to radiation. There are some studies, related to the IR drying process, reported in the literature on mint [3], pepper [20], onion slices [21], strawberry [22], kiwifruit slices, [23].

It is widely known that the drying process occurs simultaneously by different mechanisms due to the complexity of food [20]. Owing to the fact that the ability to guess the IR drying system performance, modeling of the IR heating of food products is a crucial approach in the drying industry [19].

When a detailed literature search is carried out, there is no work found on the application of IR drying on Linden leaves as a thin layer. This is a topic of pivotal importance in the literature about drying linden. The objective of the study was (1) to have an observation on the impact of drying temperature, (2) to specify the color changes and projected area changes of linden leaves, (3) to find the best convenient drying model during IR drying of linden leaves together with the effective moisture diffusion coefficients and activation energies, and (4) to find the temperature effects on total phenolic content (TPC) and total flavonoid content (TFC) of the linden leaves.

## 2. Materials and Methods

### 2.1. Materials

Linden leaves (T. platyphyllos Scop.) were harvested from the Samsun city coastline, Black Sea region, located in the north part of Turkey, particularly from the campus area of Ondokuz Mayıs University under open-air conditions. This leaf is considered one of the popular varieties in Samsun province. Its flowers have 5 petals around the flower; it is fragrant, and pale, greenish-yellow. The flower has plenty of stamens and an ovary, grouped in corymbose corymbs inflorescences, in which peduncle is partially connected to a membranous, lanceolate bract of approximately 8 cm long, rounded at the apex [7]. After the leaves collection, the samples were stored at 4 °C before drying and analysis. The destroyed and dark leaves were picked manually; only healthy structures and appearance samples were carefully selected and put into the infrared dryer as a thin layer (5 g).

### 2.2. Experimental Drying Device

**Figure 1:**
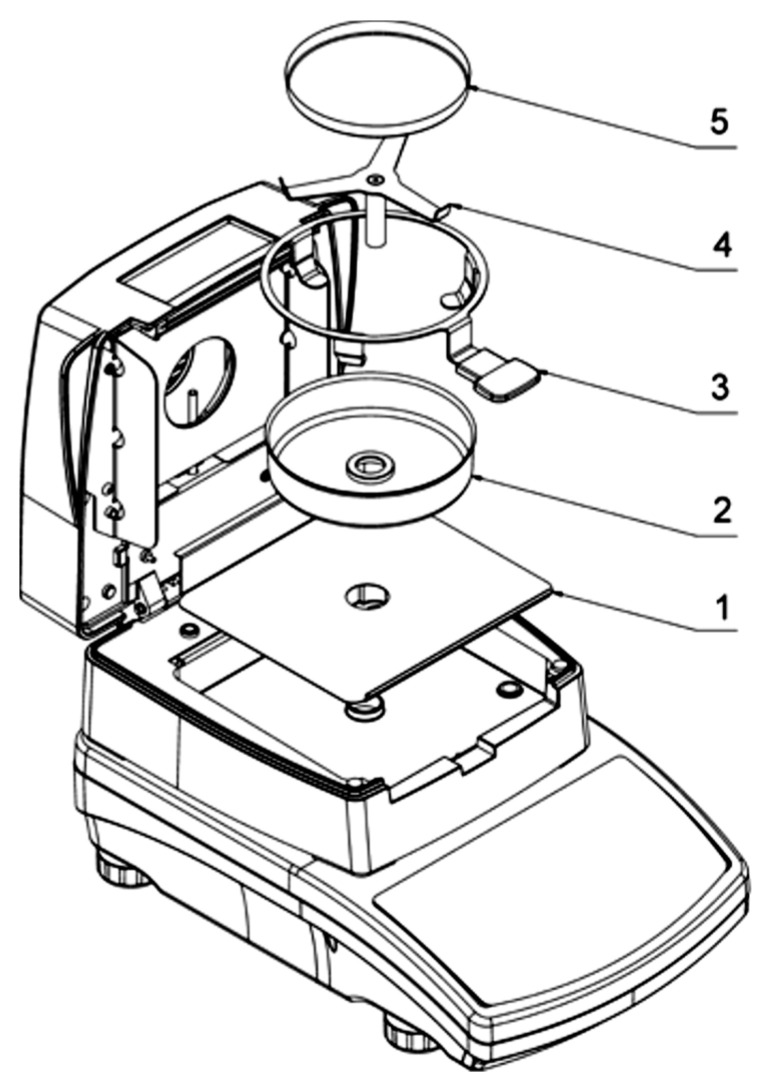
MA.R infrared (IR) dryer and moisture analyzer. 1. Drying chamber base insert. 2. Drying pan shield. 3. Drying pan handle. 4. Cross-shaped holder. 5. Disposable pan.

MA.R IR dryer and moisture analyzer (Radwag balances and scales, Warsaw, Poland) transmitting electromagnetic radiation in the range medium to shortwave IR (radiator) was used as drying equipment (Figure 1). The drying temperature was fixed in keyboard of the equipment as 50, 60, 70 °C in each experiment. In the IR drying process, the sample was distributed uniformly over the entire pan in order to prevent the reflection of the IR radiation back from the not covered area by the sample. During drying, the amount of evaporating water was designated in about 3-min intervals in each drying temperature. Trials were replicated three times and average weight loss was reported.

### 2.3. Color Measurements

**Figure 2:**
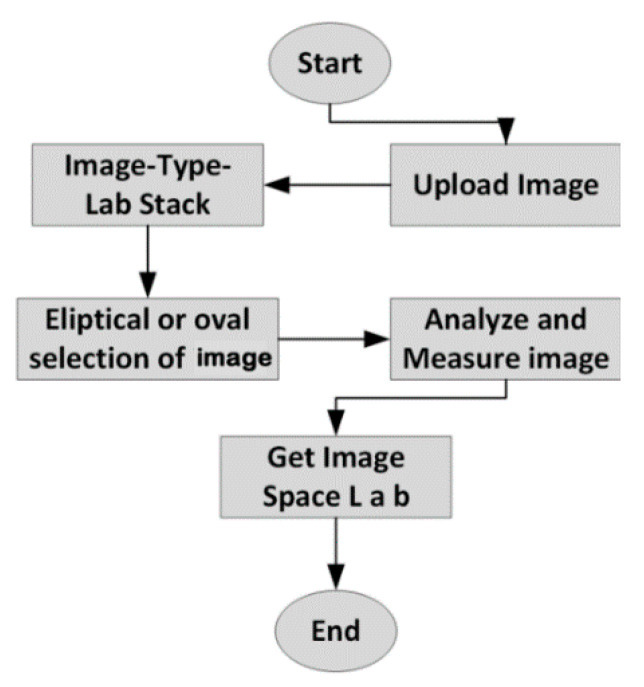
Flowchart of the ImageJ using to obtain color parameter (L*, a*, and b*). Descriptive variables (L* a* and b* ) related to color were achieved with the ImageJ software. L* variant shows the degree of lightness to darkness, a* value indicates the degree of redness (+) to greenness (−), and b* value is the degree of yellowness (+) to blueness (−) [26]. The color value of L* have shown the brightness and it ranges from 0 to 100. The color coordinates a* and b* variants do not have a particular reading range. The average values of color parameters and standard errors were calculated (L*, a*, b*, C, and H) [27].

Leaves of linden color (fresh and dried samples) were measured using the pixel method in the ImageJ program. ImageJ, developed by the National Institutes of Health (NIH), is a Java-based, readily available, open source platform, independent, and public domain software (Bethesda, Maryland USA) [24]. Before and after the drying process, this technique can be used due to being available and being free of charge to be accepted for indexed journals. The software can measure many parameters of the image analysis including the color differences, projected and surface area of leafy plants, such as linden leaves. The program uses a Lab stack to measure linden leaves. Figure 2 illustrates the flowchart using ImageJ to measure the color changing [25]. Moreover, the images were taken with the Huawei P20 16 MP camera before and after the drying process in the experiment.

C = (a^2^ + b^2^)^1/2^ (1)

h^o^ = tan^−1^ (b/a) (2)

According to Diziki, D., et al. [28], total color difference (∆E) was determined as follows:

∆E = [(L − L_o_)^2^ + (a − a_o_)^2^ + (b − b_o_)^2^]^1/2^ (3)

where L_o_, a_o_ and b_o_ indicate the brightness, redness, and yellowness of dried samples, respectively.

### 2.4. Image Analysis to Projected Area

**Figure 3:**
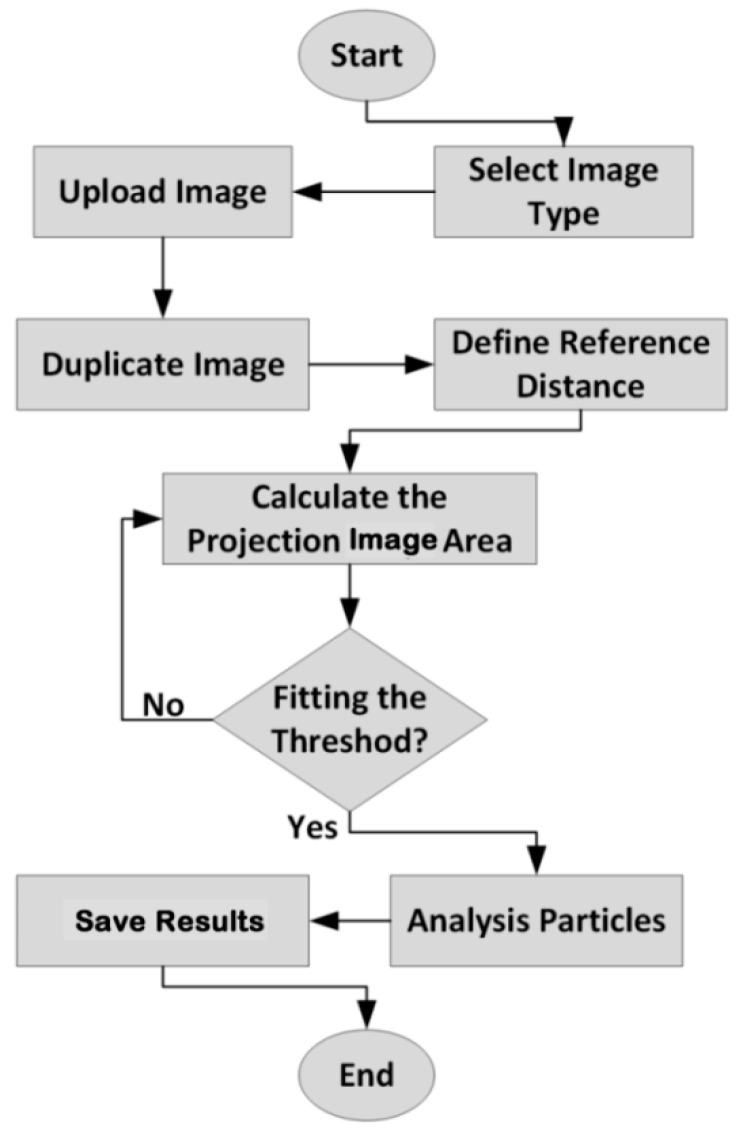
Flowchart of the ImageJ using to measure the projected area.

Image analysis can be easily applied to measure the changes of area, perimeter, and equilibrium diameter of food [29]. The determination of the projected area (PA) of thin layer linden leaves was measured using the pixel method in the ImageJ program. This method was held at two-dimensional axes (x and y) with reference to the pixel colors. The program uses a threshold-based pixel count measurement and converts their digital units (pixels) into Reference object (REF) units (cm) to calculate the leaves area [30]. Figure 3 illustrates the flowchart using the ImageJ to measure the projected area [25,26]. Nevertheless, the images were taken with the Huawei P20 16 MP camera before and after drying the process in the experiment.

### 2.5. Mathematical Modeling of Thin Layer Drying Curves

Mathematical modeling of thin-layer drying is currently the widest accepted method for investigation and optimization of dehydration characteristics in the drying process. The most frequently used moisture ratio equations were taken into account for determining the most suitable model in the thin-layer drying of Linden leaves (Table 1).

The equilibrium moisture content (*Me*) was reputed to be zero for IR drying [3] and, therefore, the moisture ratio (*MR*) was abbreviated to *M*/*M*_0_ instead of (*M* − *Me*)/*M*_0_ − *Me*); where M is the moisture content in decimal dry basis at any time t, *M*_0_ is the initial moisture content in decimal dry basis, and *M*e** is the equilibrium moisture content in decimal dry basis.

Non-linear regression analyses for these models were carried out by using SigmaPlot program (Version 12). The determination coefficient (*R*^2^), residual sum of squares (*RSS*) and standard error of estimate (*SEE*) were used as the primary parameters to select the best equation. These statistical values were calculated as follows:

(4) $$R^{2}=\frac{\Sigma_{i=1}^{N}{\left(MR_{exp,i}-MR_{pre,i}\right)}^{2}}{N-n}$$

(5) $$RSS={\left[\frac{1}{N}\Sigma_{i=1}^{N}MR_{pre,i}-MR_{exp,i}\right]}^{\frac{1}{2}}$$

(6) $$SEE=\sqrt{\frac{\sum_{1}^{N}(MR_{exp,i}-MR_{pre,i})}{N-2}}$$

where *MR_exp,i_* is the *i*th experimental moisture ratio, *MR_pre,i_* is the *i*th predicted moisture ratio, *R*^2^ is the coefficient of determination, and *N* is the number of observations [20,40].

### 2.6. The Effective Moisture Diffusivity and Activation Energy

Fick’s diffusion equation for particles with slab geometry was used for calculation of effective moisture diffusivity. General solution of Fick’s second law in slab geometry with the assumptions of moisture migration by diffusion, negligible shrinkage, constant diffusion coefficients, and temperature was as [41].

(7) $$MR=\frac{8}{\pi^{2}}\exp\left(-\frac{\pi^{2}D_{eff}t}{4L^{2}}\right)$$

(8) $$D_{eff}=L^{2}\times (-0.101\ln MR-0.0213)/t$$

where *D_eff_* is the effective moisture diffusivity (m^2^/s), L is the thickness of linden leaves (m), *t* is the time (s).

The relationship between the effective moisture diffusivity and drying temperature was described using the Arrhenius-type equation [42,43,44]:

*D_eff_* = *D*_0_ exp(−*E_a_*/(*RT_a_*)) (9)

Many researchers considered that this is a convenient and frequently used method, as well as a simplified one, and it allows calculating a constant value for the whole process. Then, taking natural logarithm of the both sides, it can be written in a linear form as follows:

ln(*D_eff_*) = ln(*D*_0_) − [(*E_a_*/*R*)(1/*T_a_*)] (10)

where *E_a_* is the energy of activation (kJ/mol), *R* is the universal gas constant (8.3143 × 10^−3^ kJ/mol), *T_a_* is the absolute temperature (K), and *D*_0_ is the pre-exponential factor of the Arrhenius equation (m^2^/s). The activation energy can be calculated from the slope of the Equation (10) by plotting ln(*D_eff_*) versus 1/*T_a_* (*K*_1_ = *E_a_*/*R*).

### 2.7. Extracts Preparation

The powdered sample (0.3 g) was extracted with methanol/distilled water (80:20, v/v) for 12 h at room temperature by the maceration method, and centrifuged for 20 min. The supernatant was used for the estimation of antioxidants and antioxidant activity.

### 2.8. Total Phenolic Content (TPC) Assay

The TPC was determined based on the Folin–Ciocalteu method [45]. A volume of 0.5 mL of the extract mixed with 2.5 mL of Folin–Ciocalteu reagent. After 5 min, 2 mL of 20% Na_2_CO_3_ solution was added with the mix, then allowed to stand for 120 min. in the dark. The absorbance was measured at 760 nm. A calibration curve was established using gallic acid as a standard, and used between 1.5–100 ppm (R^2^ = 0.9922). TPC of the samples was expressed as gallic acid equivalents (g) per 100 g dry weight (DW) (g of GAE/ 100 g DW).

### 2.9. Total Flavonoids Assay (TFC)

The TFC was measured using an AlCl_3_ colorimetric assay according to [46]. A volume of 0.25 mL of the extract was added to a test tube containing 0.75 mL of distilled water. Moreover, 0.15 mL of 5% sodium nitrite solution was added to the mix and reacted for 5 min followed by the addition of 0.3 mL of 10% aluminum chloride. After 5 min, 1 mL of 1 M sodium hydroxide solution was added. The absorbance was measured at 510 nm. A calibration curve was established using catechin as a standard. TFC was expressed as mg catechin equivalent (CE)/g of the samples.

### 2.10. Statistical Analysis

Descriptive statistics were given as average and standard error of mean. Kolmogorov Smirnov test showed that the data has normally distributed (P = 0.862) and variances were homogeneous (P = 0.534), according to the Levene test. One-Way ANOVA was used to analyze the data [47]. Power of the test found 0.976; it showed that the sample size was adequate. Duncan multiple comparison was used to compare the means tests [48].

## 3. Results and Discussion

### 3.1. Moisture Contents

**Figure 4:**
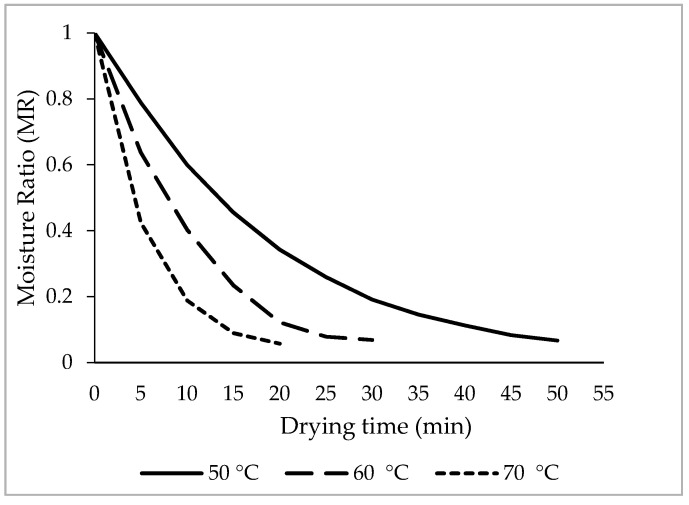
Moisture ratio changes at different air temperatures.

The moisture ratio vs. drying time at different temperatures is shown in Figure 4. The results show that the temperature had a significant effect on the drying of linden leaves. Depending on the drying temperature, the moisture content decreased to 0.10 g [H_2_O] kg^−1^ [DM] in about 20 to 50 min (Figure 4) for linden leaves. Drying time reduced dramatically with increased IR temperature. Moreover, results showed that higher drying temperature resulted in greater slope and the drying time is reduced by about 250%. The data obtained from many other studies in this field support this result [3,43,44,49,50,51]. Drying rate curves became steeper as drying temperature increased. This finding is in agreement with observations of several studies on IR drying [21,22,44].

### 3.2. Color

Indicators of fresh and dried leaves of linden at 50 °C, 60 °C, and 70 °C is presented in Table 2. The L*, a*, and b* values mentioned in the Table 2, represent lightness (L*), greenness (a*), and yellowness (b*) of linden leaves respectively.

L*, a*, and b* values of fresh linden leaves were 38.98, 1.03 and 28.81. As shown in Table 2, the L* and b* values of the dried linden leaves were increased partially, this may be due to the degradation of chlorophyll. The degree of color change was affected from oxygen level, drying temperature, and time. The magnesium in the chlorophyll structure can be replaced by hydrogen at high temperature. This displacement turns chlorophylls to pheophytins [52]. Observed a* and b* values were in the −1.84 and 3.33, 28.81, and 30.56 for dried linden leaves respectively. The a* and b* values of this study are consisted with the values found in study of Ertekin and Heybeli, ref. [31] for mint leaves. To determine the color difference, total chromatic deviation (∆E) and chromatic deviation (∆C) are not good indicators [3]. On the other hand, the resemblance of the defined colors (red, green, blue, and yellow) is called Hue (H) [53], and chrome value shows the vividness of color. Table 2 demonstrates that the color of dried linden leaves changed when the drying temperature changed. The highest Hue value was 88.81 in fresh leaves. A similar finding was found in a study by Adak et al. [22]. The limit values of ∆E were changed from 3.617 to 4.11. There were no significant differences from the point of ∆E among the temperature effects, as well as C values. Zielinska and Markowski [54] found the highest L* values at 60 °C under spout-fluidized bed drying and determined that a* and b* decreased at higher temperatures for the carrots slices.

Concerning color parameters, lightness (L*) and (a*) values increased together with temperature rise. Moreover, ∆E and C values were not affected from temperature statistically with Type I error rates (P) of 0.917 and 0.447, respectively.

### 3.3. Projected Area (PA)

**Figure 5:**
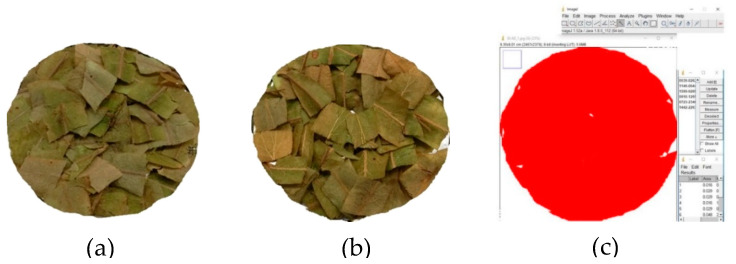
Demonstration of projected area (PA) changing at different temperatures process, (**a**) before IR drying, (**b**) after IR drying, and (**c**) calculate PA in ImageJ.

The effect of the IR process by using different temperatures on linden leaves were examined for changing of PA (Figure 5). Decreases from the PA were observed during drying of the leaves in all temperature treatments. In light of this result, it can be said that the changing of the drying temperature is the main effect on reducing the projection area of linden leaves. Statistical results (*p* < 0.05) also showed significant difference between the PA changes of dried linden leaves using IR, according to temperature (Table 3).

Depending on the temperature values (50, 60, 70 °C), the initial projection area of fresh sample leaves was 81 cm^2^. There was a 1.067 times decreasing, compared to the projection area of fresh samples. The largest PA was observed as 79.38 cm^2^ (50 °C) among treatments. At the end of the drying process, based on 50, 60, and 70 °C, the losses in the projection areas of the thin layer rose petals were 98.00%, 96.62%, and 93.74% for 50, 60, and 70 °C, respectively. The very fine structure of the leaves and the rapid removal of moisture as a result of this may have caused the shrinkage to occur at the beginning of the drying process. Briefly, this can be due to the effect of temperature on shrinkage. Similar statements (that drying reduces the PA of leaves [55]), and similar effects of drying air temperature, and time on the PA for different agricultural crops, have been reported in the literature [54].

### 3.4. Modeling of Drying Curves

For modeling purposes, the experimental moisture content data were used on the dry weight basis. These moisture content data at any time of drying process obtained under different IR drying temperatures were converted to moisture ratio values and fitted against the drying time. The 11 thin layer-drying models were compared according to their statistical parameters, such as the RSS, SEE, and R^2^ for adequacy of the model fit. The best model describing the thin layer drying characteristics of linden leaves was chosen as the one with the highest R^2^ value and lowest RSS and SEE values.

**Figure 6:**
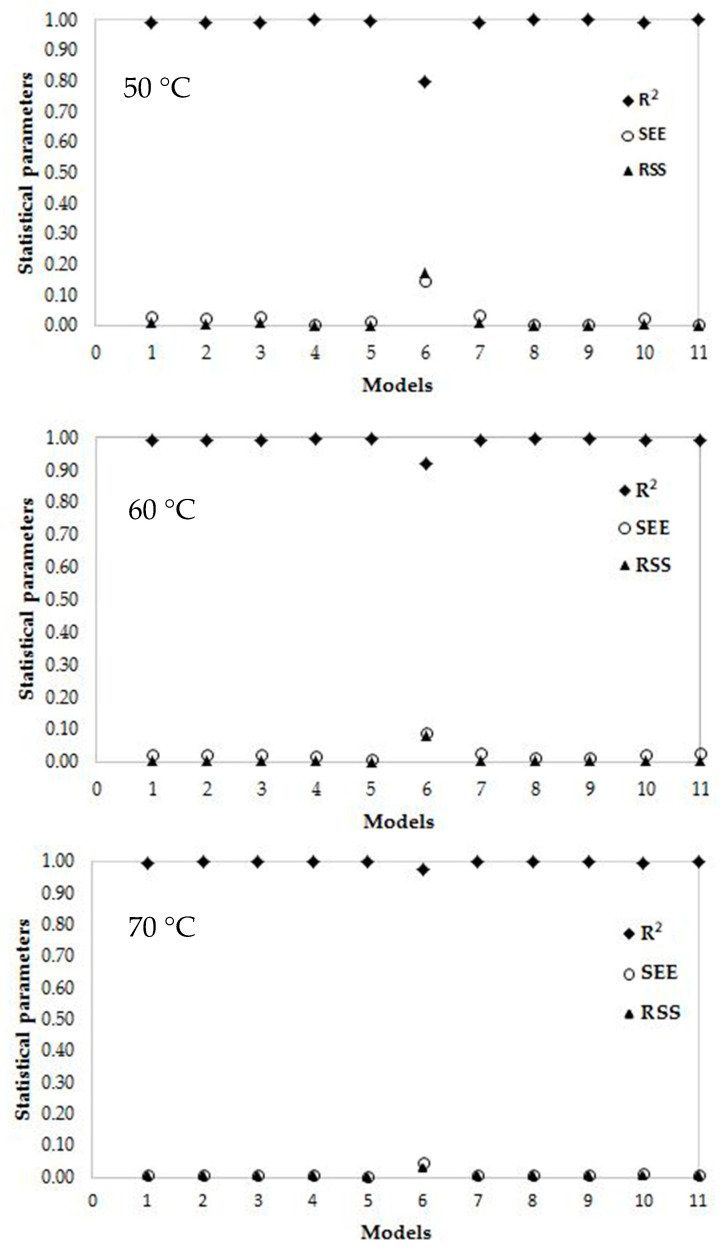
Statistical parameters of each model for different infrared (IR) drying temperatures. Where MR is the moisture ratio (dimensionless); *k*, the drying rate constant (h^−1^); *t*, the time (h) and *a*, *n* and *b* are experimental constants. The determination coefficient (R^2^), residual sum of squares (RSS) and standard error of estimate (SEE). Thus, the Midilli et al. [34] model may be assumed to represent the drying behavior of Linden leaves for 50 °C and 60 °C temperatures. It was emphasized that the Midilli et al. model [34] exhibited similar results in many other studies such as eggplant [31], kaffir lime leaves [51], carrot slices [56], Moroccan rosemary leaves [40], and pepper [20]. In addition, two- term and Logarithmic models gave relatively good results (Table 2 and Figure 6). Doymaz, I [57] surveyed four different thin-layer drying models (Lewis, Henderson, and Pabis, Modified Page, and logarithmic) and used determination coefficient, reduced R^2^, and RMSE for comparing. According to the results, logarithmic model showed a good fit than the other models.

Among the eleven drying models used in this study, the Midilli et al. [34] model gave the best results for 50 °C and 60 °C data points with values for the R^2^ greater than 0.9992, the SEE of lower than 0.0102 and the RSS of lower than 0.008 (Table 4 and Figure 6). This model was used for the linden leaves and can be shown as:

(11) $$MR=a\exp\left(-kt^{n}\right)+bt$$

On the other hand, when a comparison was made between the models for 70 °C, it was seen that the Verma et al. (R^2^ = 0.9999) and Diffusion approximation models (R^2^ = 0.9999) could represent the real values with higher accuracy than the other models. In addition, it can be said that the page model can be used if it is desired to express the drying behaviors partially with the help of a simple equation. Park et al. [58] compared Page and Fick’s model and found that Page’s model was a better fit to experimental mint drying data except for convective drying at a drying air temperature of 50 °C. In addition, the Onwude et al. study, in the pumpkin, similarly, Page model was best suited for predicting the drying kinetics of pumpkin for 60 °C, 70 °C, and 80 °C for the slices of 3 mm thickness [59]. As a result, the four different models, especially the Midilli et al model [34], predicted well the moisture ratio (MR) at three drying temperatures 50 °C, 60 °C, 70 °C for the linden leaves.

### 3.5. Effective Moisture Diffusivity and Activation Energy

The drying process of linden leaves can be easily modeled as a diffusion process as the entire drying process occurs in the falling rate period. Table 5 shows the effective moisture diffusivity (*D_eff_*) and activation energy (*E_a_*) values for linden leaves under different drying temperatures.

**Figure 7:**
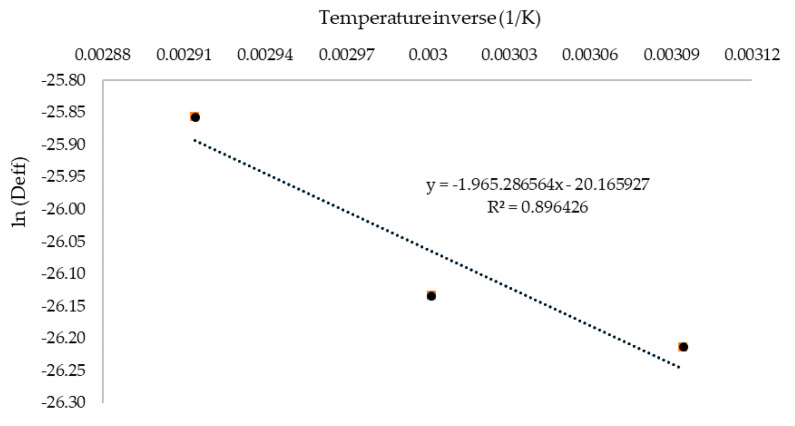
ln*D_eff_* versus absolute temperature inverses (T).

The *D_eff_* values varied from 4.13 × 10^−12^ to 5.89 × 10^−12^ m^2^/s and over the temperature range studied for biological materials [60]. According to Hii et al. [60] and Zogzas et al. [61], the values of moisture diffusivity of food products lies in the range of 10^−7^ to 10^−12^ m^2^/s. The variations in the present study might be because of *D_eff_* is substantially hinged on drying conditions, features of drying equipment, recommended models used for calculation, product specifications as physico-chemical properties, such as compound, texture characteristics, layer thickness, cultivar and ripening stage, and other uncontrolled parameters [17,62]. The observations also agree with the results reported by Abraham et al. [63], Guine et al. [64], and Madan et al [65]. Noteworthy rises were acquired in the effective moisture diffusivity values with the increase in IR drying temperature (*p* < 0.001). This was because higher drying temperatures expedites the water molecules in the product and, as a result, rapid moisture loss occurs at higher temperatures as compared to lower temperatures. Moreover, rapid decline of the product moisture content corresponds toa higher value of effective moisture diffusivity [66]. The logarithm of *D_eff_* as a function of bivious of absolute temperature (T) is also demonstrated in Figure 7. The results show a linear relationship between (ln*D_eff_*) and (1/T) or an Arrhenius-type relationship.

On the other hand, the diffusivity constant in other words “pre-exponential factor” of the Arrhenius equation (*D*_0_) was predicted as 1.746 × 10^−09^ m^2^/s for linden leaves. Activation energy (*E_a_*) was also estimated using exponential regression as a 16.339 kJ/mol for the linden leaves (Table 5).

This result is within the range of 15–40 kJ/mol for various foods [20]. When activation energies of fruits and vegetables are evaluated, more than 90% of the activation energy values that were found in previous studies ranged between 14.42 and 43.26 kJ/mol, for example; 28.60 kJ/mol for bamboo [66], 38.34 kJ/mol for sliced lemongrass [44], 28.36 kJ/mol for thin layer carrot [67]. The activation energy of present study for linden leaves is relatively low. The *E_a_* value shows the sensitivity of diffusivity against temperature [68]. This means that the lower the *E_a_* values, the less sensitivity of the diffusivity to the temperature and, so, a lower value indicates high moisture diffusivity [67]. Hence, in the present study, about 16.339 kJ/mol of energy is required for the moisture diffusion and subsequent evaporation from the surface of the leaves.

### 3.6. Total Phenolic Content (TPC) and Flavonoids (TFC)

The results are given by calculating on dry matter values to prevent errors arising from dry matter difference. The total phenolic content (TPC) of fresh leaves material was significantly (*p* < 0.05) higher than dried leaves. Similarly, some recent searches have signified that dried plant materials include higher polyphenolics as antioxidants compared to fresh plant materials [68]. Table 6 presents the TPC and TFC content of the linden leaves under different temperatures process.

To analyze the data, non-parametric permutation test was used because of heteroscedasticity [69]. TPC and TFC values were corrected and evaluated based on dry matter values to prevent errors arising from dry matter differences. Table 6 shows that, TPC of linden leaves were significantly different between fresh and dried samples and the values ranged from 99.756 ± 0.63 mg/g to 127.73 ± 0.76 mg/g. The TPC in the dried leaves (for 50 °C, 60 °C, 70 °C) were significantly (*p* <0.001) lower than that in the fresh. TPC decreasing after IR drying may be caused by enzymatic processes. Moreover, a noteworthy decrease of total phenols concentration between fresh and dried samples, probably by the reason of generation of different antioxidant compounds having a varying degree of antioxidant activity. The same results were observed by Rababah, Al-u’datt, Alhamad, Al-Mahasneh, Ereifej, Andrade, Altarifi, Almajwal, and Yang [5] for sage, lemon balm, and thyme; Lopez et al. [70] for blueberry. When they evaluated the content of phenolic compounds after drying, higher TPC values in the fresh leaves than in the dried samples it have been found. It has also been reported by Felipe et al. [71] that the drying process can cause up to, approximately, a 30% decrease in total phenol content.

On the other hand, as can be seen in Table 6, the Duncan test indicates no statistical difference among temperatures (50 °C, 60 °C, and 70 °C). This means that linden leaves seem to be thermostable in the studied temperature range. This may indicate that linden leaves are thermostable in terms of the phenolic components they contain. Based on the drying process, similar observations were reported by Zlotek et al. [72]. Moreover, some researchers proposed that—not only the amount of antioxidants, but also a synergy occurring between them and the other leaves—ingredients might influence the differences in the antioxidant ability of material extracts [73].

Similarly, when the TFC were evaluated, a tendency to be directly proportional to the phenol contents was observed. The TFC in linden leaves are shown in Table 6; it varied significantly between fresh and dried samples and ranged from 0.567 ± 0.015 mg/g to 2.790 ± 0.150 mg/g. The reason for this may be the decrease of the solution viscosity due to the increase in temperature as the lime leaves change from a wet state to dry state, and the increase of solubility, accordingly [74]. Roshanak et al. [75] reported the high amount of TFC in dried green tea compared to fresh samples. In another study, Azad et al. [76] examined the effects of the IR drying process on Angelica gigas Nakai Powder and found that there was a decrease in total flavonoid content due to the temperature increase compared to the fresh sample. On the other hand, although the greatest TFC value among temperature applications was achieved at 50 °C, no statistically significant change (<0.001) was observed in the values due to the increase in temperature. As with the total phenol content values, the TFC values remained stable due to the temperature increase. The stability of the flavonoids depends on the type and number of substituents; sugar moiety and methoxyl groups protected flavonoids from different drying processes, such as IR, microwave, ultrasonic-induces degradation, hydroxyl groups, and the presence of non-phenolic compounds promoted it [77]. In this study, depending on the type of flavonoids and the number of substituents, there could be no change in the flavonoid contents. In addition, the TFC results obtained in the present study correlated with Olsson et al. [78]. They reported that heating has no causes—significant differences—in total flavonol content in sweet cultivars and in red onion cultivars.

Drying treatments have various effects on TPC and TFC, as stated by these observations. This finding suggests that besides the Midilli drying model, a simpler Page model can also be preferred for linden leaves under an IR thin layer drying process. In addition, 50 °C will be sufficient in terms of phenol content and flavonoids content in a thin layer lime leaf drying process with IR. In terms of energy saving, higher temperatures, such as 60 °C or 70 °C, can be avoided. The functional properties of food drying that influenced were by the intricate chemical interactions are still under research.

## 4. Conclusions

This study investigated the potential of using the thin layer as a modeling tool for predicting the drying process of linden leaves samples. The performance of IR drying technique for drying linden leaves samples was evaluated. This study also identified changing of color parameters, PA, and a suitable mathematical model to describe the behavior of thin layer linden leaves subject to IR drying. In addition, the moisture diffusion coefficient and the activation energy of the linden leaves were also estimated. Experiments for linden leaves were managed at specific drying temperatures of 50 °C, 60 °C, 70 °C.

The results showed that significant changes in color values occurred due to temperature change. The highest color parameters were achieved at 60 °C, except a* and H values. ∆E was not significantly affected from temperature change. The PAs of the dried linden leaves decreased 98.00%, 96.62%, and 93.74% for 50, 60, and 70 °C respectively. Moreover, the findings demonstrate that higher drying temperature is associated with shorter drying time and faster moisture removal rate. Drying time is reduced by about 250% with the effect of the IR process under different temperatures. The Midilli model (R^2^ = 0.9999) was found to be the most suitable model describing thin-layer drying by IR for 50 °C, 60 °C, while the Verma et al. (R^2^ = 0.9999) and Diffusion approximation models (R^2^ = 0.9999) for the 70 °C. Effective moisture diffusivity varied from 4.13 × 10^−12^ to 5.89 × 10^−12^ over the temperature range studied, with activation energy of 16.339 kJ/mol.

Moreover, the effects of temperature on TPC and TFC by IR drying demonstrated that both values increased together with increasing temperatures. Drying temperature of 50 °C degrees in the thin layer IR drying process for these type of linden leaves could be recommended in terms of TPC and TFC. In addition, considering the further investigation in terms of TPC, more detailed results could be obtained with HPLC analysis instead of the Folin–Ciocalteu method. In this study, three different temperature effects on color, PA, some drying models, TPC, and TFC values were evaluated. Further study is recommended to understand the moisture diffusion mechanism, and any other properties changing at different drying processes, together with IR for linden leaves.

## Figures and Tables

**Table 1:** Mathematical models applied to the drying curves in the study.

	Model Name	Model Equation	References
1	Newton	MR=exp(−kt)	[31]
2	Page	$MR=\exp\left(-kt^{n}\right)$	[32]
3	Henderson and Pabis	MR=aexp(−kt)	[31]
4	Logarithmic	MR=aexp(−kt)+b	[33]
5	Midilli et al.	$MR=a\exp\left(-kt^{n}\right)+bt$	[34]
6	Wang and Singh	$MR=1+at+bt^{2}$	[35]
7	Logistic	MR=b/(1+aexp(kt))	[36]
8	Two term	$MR=a\exp\left(-kt\right)+b\exp\left(-k_{1}t\right)$	[36]
9	Verma et al.	MR=aexp(−kt)+(1−a)exp(−bt)	[37]
10	Two term exponential	MR=aexp(−kt)+(1−a)exp(−kat)	[38]
11	Diffusion approximation	MR=aexp(−kt)+(1−a)exp(−kbt)	[39]

*MR*: moisture ratio; *k* and *k*_1_: drying coefficients; *n*: exponent; *t*: time in min; *a* and *b*: coefficients.

**Table 2:** Color indicators of linden leaves at different temperature.

	L*	a*	b*	C	H	ΔE
Fresh	38.98 ± 1.05 ^b^	1.03 ± 0.11 ^b^	28.81 ± 0.53 ^a^	28.83 ± 0.54 ^a^	88.81 ± 0.61 ^a^	-
IR-50 °C	38.86 ± 0.66 ^b^	−1.84 ± 0.02 ^c^	29.03 ± 0.86 ^a^	29.09 ± 0.86 ^a^	−86.36 ± 0.14 ^c^	3.617 ± 0.20
IR-60 °C	42.36 ± 0.63 ^a^	1.22 ± 0.06 ^b^	30.56 ± 0.35 ^a^	30.59 ± 0.35 ^a^	88.34 ± 0.66 ^a^	4.11 ± 1.32
IR-70 °C	40.95 ± 1.11 ^ab^	3.33 ± 0.10 ^a^	29.63 ± 1.18 ^a^	29.81 ± 1.17 ^a^	83.4 ± 0.93 ^b^	3.81 ± 0.54
*p*-value	0.047	<0.001	0.455	0.447	<0.001	0.917

L*, a*, and b* values represent fresh linden leaves; The resemblance of the defined colors (red, green, blue, and yellow) is called Hue (H); Chromatic deviation (C); Total chromatic deviation (∆E); Different letters in same column indicate statistical difference (*p* < 0.05).

**Table 3:** Projection area changing of linden leaves under IR processing at different temperatures.

	Projection Area (cm^2^)
Fresh	81.00 ± 0.00 ^a^
50 °C	79.38 ± 0.14 ^b^
60 °C	78.26 ± 0.09 ^c^
70 °C	75.93 ± 0.14 ^d^
*p*-value	<0.001

Different letters in same column indicate statistical difference (*p* < 0.001).

**Table 4:** Statistical models and its constant values for the thin layer drying models.

Temperature °C	Statistical Model and Its Constants						
	Midilli et al. Model						
	*R* ^2^	*SEE*	*RSS*	*k*	*n*	*a*	*b*
50	0.9999	0.0025	0.0001	0.0413	1.0933	0.9998	0.0003
60	0.9992	0.0102	0.0008	0.0716	1.1305	0.9984	0.0009
70	0.9984	0.0152	0.0014	0.1300	1.1340	0.9914	0.0013
	Verma et al. model						
50	0.9991	0.0095	0.0011	−0.1453	-	6.64 × 10^−7^	0.0534
60	0.9978	0.0159	0.0023	−0.0567	-	0.0020	0.0963
70	0.9999	0.0041	0.0001	−0.0121	-	0.0267	0.1814
	Diffusion approximation model						
50	0.9994	0.008	2.4640	2.1067	-	−0.0385	0.0262
60	0.9936	0.0269	0.0065	1.4003	-	−0.0229	0.0682
70	0.9999	0.0041	0.0001	0.1814	-	0.9733	−0.067
	Page model						
50	0.9992	0.009	0.0011	0.0472	1.0381	-	-
60	0.9935	0.0257	0.0066	0.0955	0.9915	-	-
70	0.9931	0.0269	0.0058	0.2584	0.7815	-	-

The determination coefficient (R^2^), residual sum of squares (RSS) and standard error of estimate (SEE); *k*, the drying rate constant (h − 1), *a*, *n* and *b* are experimental constants.

**Table 5:** *D_eff_* coefficients and *E_a_* of linden leaves under different IR drying temperatures.

Drying Temperature (°C)	*D_eff_* (m^2^s^−1^)	*D*_0_ (m^2^s^−1^)	*E_a_* (kJ/mol)
50	4.13 × 10^−12^	1.746 × 10^−09^	16.339
60	4.47 × 10^−12^		
70	5.89 × 10^−12^		

*D_eff_* stands for the effective moisture diffusivity, *D*_0_ is the pre-exponential factor of the Arrhenius equation (m^2^/s) and *E_a_* is activation energy.

**Table 6:** Total phenolic and flavonoids content of the fresh and dried linden leaves.

Temperature (°C)	TPC (mg/g, DW)	TFC (mg/g, DW)
Fresh	127.73 ± 0.76 ^b^	0.567 ± 0.015 ^b^
50	95.184 ± 0.47 ^a^	2.790 ± 0.150 ^a^
60	99.756 ± 0.63 ^a^	2.631 ± 0.084 ^a^
70	98.929 ± 0.43 ^a^	2.583 ± 0.145 ^a^
Significance	<0.001	<0.001

The total phenolic content (TPC), total flavonoid content (TFC), a, b: Different letters within same column shows the statistical difference (*p* < 0.01).

## Abbreviations

C—degree (°), PA—projected area (mm^2^), TPC—total phenolic content (mg/g), TFC—total flavonoid content (mg /g), L*—lightness, a*—redness, b*—blueness, C*—chrome, H*—hue angle, ∆E—color difference, *D_eff_*—effective moisture diffusivity (m^2^s^−1^), *E_a_*—activation energy (kJ/mol).
